# Editorial Correspondence

**Published:** 1877-01

**Authors:** 


### EDITORIAL CORRESPONDENCE.

MEDICAL ASSOCIATION OF GEORGIA—A REPLY to "A COUVERT M. D."

*Mr. Editor:*

As a member of the State Medical Association, I beg to make a brief reply to the letter of "A. Couvert, M. D.," addressed, through your November number, to the President and members of the Association. If the writer had been familiar with the relative proportion of the paying and non-paying membership, he might have hesitated in so lavish an expenditure of so much really valuable sarcasm.

I beg to present several facts and opinions:

1st. The Association has between four and five hundred members. Of these fifty-two (52) have paid this year's assessment—about the usual average.

2d. Five dollars is too large an amount for the annual assessment, with so large a membership.

3d. It is not right that a few should pay that amount, and others nothing, year after year.

4th. Settlement of dues can be enforced only by a penalty. The only available penalty is a forfeiture of membership.

5th. Those who have paid have just cause of complaint that the Association requires them to support the organization, rather than lighten their burden by enforcing at least a reasonable compliance with the rules on the part of all.

6th. Enforcement of the by-laws will secure settlement from all who really desire to retain their connection with the body. Those who have no such desire will possibly not wait for the society to strike their names from the list.

7th. The assessment can be easily reduced—and, doubtless, will be—to two dollars, or less, by adherance to the rules recently established.

8th. While the assessment, for the present, is five dollars per year, membership is not forfeited unless the member has failed for *two consecutive* years to settle his dues.

Now, I dare say, "A. Couvert, M.D." is one of the "gallant fifty-two," and I, with him, raise my voice—but, of necessity, in a less sarcastic strain—and proclaim my woes; and I ask the Association to enforce the rules—as other societies do—in order that all of us may retain our membership without being subjected to burdensome assessment in the future.

Very respectfully,

JNO. THAD. JOHNSON.

ATLANTA, GA., November, 1876.

---

WATERPROOF, LA., Jan. 2, 1877.

*Messrs Editors:*

I adopt the following plan for the management of hemorrhagic malarial fever:

This is a disease, so far as my knowledge of it goes, which succeeds long attacks of intermittent fever. It is ushered in by a chill; fever follows; the skin turns yellow, and the patient micturates bloody urine. The disease may be intermittent or remittent in its form; but in all the cases I have seen I have never met but one of the intermittent type. In this case, a boy eight years old, the chill came on between eleven and twelve o'clock A.M., and the fever would go off toward night. The boy rested well, and would want to get up the next morning and play with the rest of the children. Between the paroxysms the patient voided no bloody urine. It seemed to be normal.

The treatment was quinine, as a prophylactic against the recurrence of a paroxysm, and cathartics when indicated. The patient was kept on this treatment for six days. After that date I had my brother, Dr. J. S. Pugh, in consultation with me, and we changed the prescription to iron, gentian, and taraxacum. The boy had no more fever, no more bloody urine, and his recovery was rapid and permanent.

The next case I had was a little girl, aged eleven years. She was threatened with a chill, and her mother had me to give her a decided dose of quinine. In two hours time I was called back to see the girl, whom I found with a perfect case of hemorrhagic fever. At that time I regarded quinine, together with mild chloride, as the "sheet anchor" in this malady, and therefore gave them in full and repeated doses. This patient did as I believe all others will do who are treated on that plan. With six years experience in the treatment of this disease, I have concluded that calomel and quinine are not the "sheet anchors." I believe I have now adopted a more suc cessful plan of treatment in this disease, which is as follows:

I direct—

| | | |
|---|---|---|
| ℞.—Wine of Ergot, | . . . . | 60 drops. |
| Muriated Tr. Iron, | . . . . . | 20 drops. |
| Oil Turpentine, | . . . . | 5 drops. |
| Spirit Nitre Dulc., | . . . . . | 20 drops. |

Mix and give all at once, in a gill of water.

I have this prescription repeated every two hours until the urine clears up. After this I leave off the ergot and oil turpentine, but continue iron and nitre, and add to them a preparation of nux vomica—the fluid extract—in five-drop doses, every four hours, until I am sure my patient is safe from a recurrence of the disease, and then direct it every eight hours, or before meals. The danger in this disease is not from an excessive flow of bloody urine, but rather from a suppression of urine.

Some physicians put but little faith in iron in the treatment of this disease, but, I am of opinion, without a proper knowledge of the pathology of the disease. We have general anæmia. The muscular system is in a relaxed condition. The circulatory apparatus is in a like state, and the blood is depressed and abnormal. Some use calomel in small doses, in order that they may produce slight ptyalism. They believe that if they can produce salivation their patient is safe. I have held to this opinion myself, but now think we can dispense with calômel, unless there be a sure and positive indication for its use. Some use belladonna, and speak favorably of its influence in controling the hæmaturia. I do not think it hurtful, and there can be no objection to using it when other better agents are not at hand. If the stomach is irritable, as it is most apt to be, I have found hypodermic injections of morphine, together with the effervescing draught, or lime water and milk, to control that difficulty.

With the sincere hope that my views in regard to this intractable disease may be beneficial to the profession and those who suffer with it,

I am, very truly,

Thos. J. Pugh, M.D.

